# Evaluation of the safety of PD‐1/PD‐L1 inhibitors for immunotherapy in patients with malignant tumors after COVID‐19 infection: A single‐center cohort study

**DOI:** 10.1002/cam4.70202

**Published:** 2024-10-08

**Authors:** Kaili Liao, Jinting Cheng, Yujie Hu, Beining Zhang, Peng Huang, Jie Liu, Wenyige Zhang, Huan Hu, Xinyi Bai, Yihui Qian, Daixin Guo, Kun Ai, Yuchen Zhu, Long Huang

**Affiliations:** ^1^ Department of Clinical Laboratory, the 2nd Affiliated Hospital, Jiangxi Medical College Nanchang University Nanchang China; ^2^ School of Public Health, Jiangxi Medical College Nanchang University Nanchang China; ^3^ The 1st Clinical Medical College, Jiangxi Medical College Nanchang University Nanchang China; ^4^ Queen Mary College, Jiangxi Medical College, Nanchang University Nanchang China; ^5^ Department of Oncology, the 2nd Affiliated Hospital, Jiangxi Medical College Nanchang University Nanchang China; ^6^ The 2nd Clinical Medical College, Jiangxi Medical College Nanchang University Nanchang China

**Keywords:** cancer, COVID‐19 infection, immune‐related adverse events, PD‐1/PD‐L1 inhibitor, propensity score matching

## Abstract

**Introduction:**

An increasing body of evidence suggests a close association between COVID‐19 infection and the safety of PD‐1/PD‐L1 inhibitor therapy in cancer patients. However, the available data concerning these impacts remain limited and occasionally contradictory.

**Material and Methods:**

We conducted a retrospective analysis of cancer patients who received PD‐1/PD‐L1 inhibitor therapy at the same institution from November 2022 to May 2023. After excluding patients with missing information, a total of 224 cases were included. In our study, immune‐related adverse events (irAEs) that occurred during the hospitalization of patients were included in the analysis. Further analysis of inter‐subgroup differences was conducted following a 1:2 propensity score matching. Statistical analyses were performed using the Fisher's exact, chi‐squared, and Mann–Whitney *U*‐tests.

**Result:**

The results showed that no statistically significant differences between the two subgroups in the incidence of irAEs, changes in immune function before and after using PD‐1/PD‐L1 inhibitors, and alterations in hepatic and renal function (*p* > 0.05).

**Conclusion:**

Our findings suggest that infection with COVID‐19 does not significantly impact the safety of PD‐1/PD‐L1 inhibitors in cancer patients. Most cancer patients used PD‐1/PD‐L1 inhibitors during COVID‐19 infection (asymptomatic or mild infection) did not experience exacerbation of their underlying condition, nor did they exhibit a substantial increase in toxic side effects.

## Graphical abstract

Investigative protocol for conducting a single‐center cohort study to evaluate the safety of PD‐1/PD‐L1 inhibitors for immunotherapy in patients with malignant tumors after COVID‐19 infection.

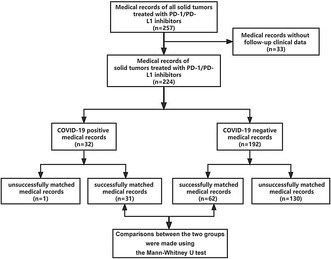

## 1 INTRODUCTION

Tumor immunotherapy is increasingly becoming the future direction of tumor treatment, with PD‐1/PD‐L1 inhibitors leading the way in recent years. PD‐1 functions as a suppressive factor on the surface of T cells, while PD‐L1 is excessively expressed on malignant tumor cells' surface. Subsequently, PD‐L1 forms pairs with PD‐1 to hinder the proliferation and activation of T cells. This results in the deactivation of T cells and ultimately leads to immune evasion, causing immunotherapy to be ineffective.^1^ The pathway involving PD‐1/PD‐L1 is of utmost importance in the field of tumor immunotherapy, and its inhibitors have achieved significant advancements in therapy, providing advantages to numerous individuals suffering from cancer.^2^, ^3^, ^4^, ^5^ However, owing to the global pandemic of novel coronaviruses, several therapeutic decisions for cancer patients have undergone significant changes.

Immunological studies of patients recovering from COVID‐19 revealed a depletion of T cells and an increase in PD‐1^+^ T cells. The patient's plasma levels of IL‐1β, IL‐1RA, IL‐8, and other substances also showed an increase. This modified immune phenotype is reflected in diminished and impaired ex vivo and in vivo T‐cell responses to both non‐specific and specific stimuli, including reduced responses to SARS‐CoV‐2 antigens.^6^ Moreover, the infection of lung epithelial cells by SARS‐CoV‐2 leads to the generation of inflammatory cytokines like IL‐6 and TNF‐α. These cytokines also induce an increase in PD‐L1 expression in infected epithelial cells and disrupt the regulation of PD‐L1 in different immune cells.^7^ These findings have significant clinical implications and offer a fresh rationale for utilizing PD‐1/PD‐L1 inhibitors in COVID‐19‐infected patients.^8^

However, there is limited and conflicting data on the impact of COVID‐19 infection on cancer patients who are undergoing PD‐1 immunotherapy. The safety of PD‐1/PD‐L1 inhibitors in the presence of COVID‐19 infection has become a crucial concern for oncologists and cancer patients. In a prior investigation, Ghosh et al. systematically evaluated the modifications in the utilization of immune checkpoint inhibitors (ICIs) among individuals registered in a prospective database for rheumatic immune‐related adverse events (irAEs) during the height of the COVID‐19 outbreak. The results indicated that cancer patients with ICI rheumatic irAEs may be more susceptible to COVID‐19, but it does not necessarily worsen the disease.^9^ In a separate study, findings indicated that the standard procedure of prescribing and administering ICI in Italy remained unaffected by the COVID‐19 pandemic, with telemedicine continuing to be extensively utilized.^10^ Meanwhile, a large multicenter study by Mei et al. investigated the effects of COVID‐19 vaccination on PD‐1 inhibitor therapy in cancer patients. The study analyzed 579 patients with 15 different types of cancer and revealed that no serious side effects resulted from COVID‐19 vaccination, and no serious anti‐PD‐1‐related adverse events (irAEs ≥3) were reported.^11^ Luo et al. discovered that the risk of COVID‐19 severity was not increased by PD‐1 blockade exposure, even after accounting for smoking status. They also found that the severity of COVID‐19 in lung cancer patients remained unaffected by PD‐1 blockade. This determination was derived from an examination of a study group comprising 69 individuals diagnosed with lung cancer who fulfilled the required qualifications.^12^ However, it is essential to note that this study only focused on a single type of cancer, and further evidence is required to support these findings.

The above information inspired our study. We focused on patients with multiple types of cancer who had undergone PD‐1/PD‐L1 inhibitor therapy and analyzed the number of irAEs events and the different types of irAEs in the entire group. To determine if there was a significant association between the quantity and nature of irAEs and COVID‐19 infection, We conducted propensity score matching (PSM) analyses on patients' age, gender, cancer stage/metastasis, underlying disease, cancer type, PD‐1/PD‐L1 inhibitor type, and ECOG. Subsequently, we assessed if there were significant variations in the occurrence of irAE events among the subgroups. To further evaluate the influence of COVID‐19 infection on the safety of drug usage, we also examined patients' blood, liver, and kidney function data before and after the administration of the drug. In this single‐center retrospective cohort study, our statistical analysis demonstrated that COVID‐19 infection did not significantly influence the occurrence of irAEs, changes in immune system function, or changes in hepatic and renal function in cancer patients using PD‐1 inhibitors. This study provides deeper insights into the safety of PD‐1/PD‐L1 inhibitor usage and serves as a valuable clinical reference for patients receiving immunotherapy.

## 2 METHODS

### 2.1 Inclusion criteria

We collected 257 oncology cases that received PD‐1/PD‐L1 immunosuppressants between November 9, 2022, and May 9, 2023, in the Second Affiliated Hospital of Nanchang University. We excluded cases that lacked follow‐up clinical data and finally sorted out 224 cases. This single‐center retrospective study was approved by the Medical Ethics Committee of the Second Affiliated Hospital of Nanchang University. According to the respective tumor type, the tumor stage of the patients in the case follows the TNM, FIGO, BCLC, or CNLC staging criteria, ranging from stage I to IV and stage A to stage C. During this period, patients in each case underwent regular nucleic acid tests in the hospital, and the ECOG PS score was ≤2, indicating that the patient's overall health was relatively good. Cases were grouped into COVID‐19 positive and negative cohorts. Participants eligible for the positive cohort met the following inclusion criteria: (1) Patients should be over 18 years old; (2) The test result is positive in the hospital nucleic acid test; (3) Each patient tested positive during PD‐1/PD‐L1 immunosuppressive therapy; (4) The ECOG PS score of the selected patient should be ≤2. In the end, 32 new COVID‐19‐positive cancer patients met the requirements.

### 2.2 Data collection

We manually collected personal information, clinical information, and medical history of the medical records when they were included in the trial and followed up on treatment protocols, adverse events, and outcomes from the current immunotherapy to the subsequent immunotherapy until May 30, 2023. All recorded irAEs were new during the course of treatment and there were no long‐standing immune adverse events from prior treatment. We also counted patients on combination therapy. Clinical information includes blood routine, liver function, kidney function, myocardial enzyme, interleukin‐6, erythrocyte sedimentation rate, BNP, blood oxygen, and other test data before and after the use of PD‐1/PD‐L1 immunization agents, as well as the regimen and cycle of immune drug use. We used the United States Eastern Oncology Collaboration (ECOG) criteria to assess patient functional status.

### 2.3 Patient grouping and data analysis

**FIGURE 1:**
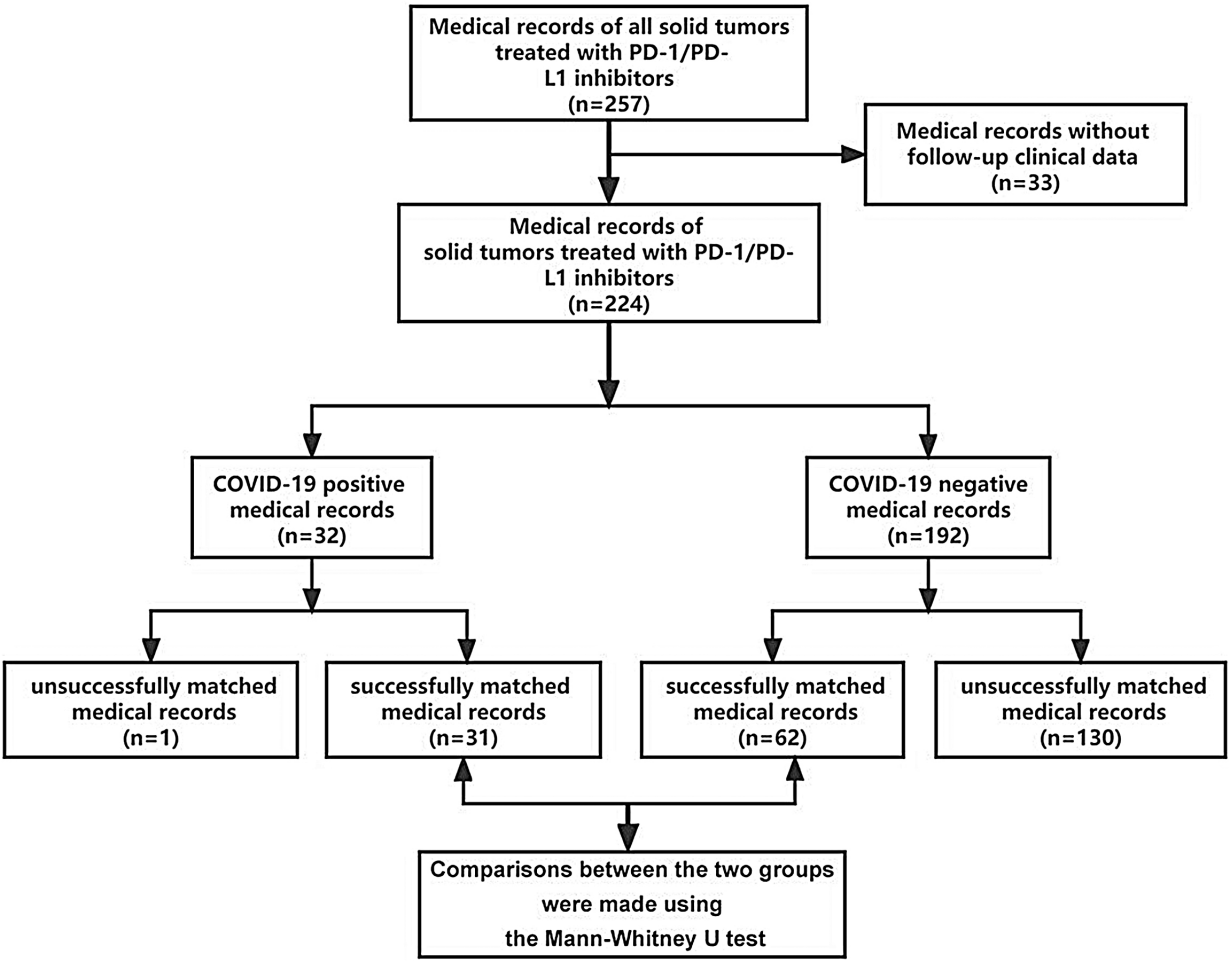
Flow diagram of the patient cohort.

Baseline data analysis was performed for all 224 cases included. Baseline data variables included sex, tumor stage, ECOG score, type of cancer, underlying diseases, type of PD‐1/PD‐L1 immunosuppressant, and number and type of immune‐related adverse reactions. The cases were categorized into groups based on their COVID‐19 test results. Categorical variables were presented as *n* (%), and statistical tests such as Fisher's exact test and chi‐square test were employed to compare characteristics among different subgroups. Continuous variables were expressed as median (interquartile distance), and the Mann–Whitney *U* test was used for comparison between groups. To compare the impact of COVID‐19 infection on the safety of PD‐1/PD‐L1 immunization in cancer patients, R‐3.5.3 was used in this cohort according to age, sex, cancer stage/type, underlying disease, type of PD‐1/PD‐L1 inhibitor and ECOG. In order to compare the impact of COVID‐19 infection on the safety of PD‐1/PD‐L1 immunization in cancer patients, we used PSM of R‐3.5.3 and SPSS‐26 (Install the R Plug‐in for Statistics and PS Matching) to match the COVID‐19 positive group with the COVID‐19 negative group by 1:2 according to age, sex, cancer stage/type, underlying disease, PD‐1/PD‐L1 inhibitor type, and ECOG in the cohort. This method uses the propensity score of individuals to match, and propensity score is obtained by logit regression, and as many covariates as possible are selected, which is similar to control variables in regression and match objects according to propensity score. We performed the same baseline data analysis for 31 successfully matched subgroups. We also used GraphPad Prism 8.0.2 to analyze the differences in blood routine and liver and kidney function data before and after the use of PD‐1/PD‐L1 immunosuppressants in these 31 matched subgroups of patients and presented them in the form of scatter plot and violin plot. Finally, we conducted the Mann–Whitney *U* test for the differences in the above clinical information between the two subgroups. In addition, we also performed matching analyses of different cancer types in patient cohorts of lung, liver, and gastric cancers, respectively, and used Fisher's exact test to compare the covariate characteristics between subgroups after successful matching as well as the differences in the number and types of immune‐related adverse reactions between the two groups. All *p*‐values were double‐tailed, and *p* < 0.05 was statistically significant. The above related process is shown in Figure 1.

## 3 RESULTS

### 3.1 Basic patient characteristics

A total of 224 cases were included in this study, with 32 cases (14.3%) in the COVID‐19 positive group and 192 cases (85.7%) in the COVID‐19 negative group. The baseline statistics of the patients are shown in Table 1. The age range of the entire cohort was 28–81, with a median age of 59, and there was a total of 179 cases (79.9%) of males and 45 cases (20.1%) of females. The most common type of cancer in the entire cohort was liver cancer, with a total of 70 cases (31.3%), followed by cancers of the digestive system (27.7%), lung (25.4%), nasopharyngeal (3.1%), and urologic (2.2%), with the remaining other types of cancer totaling 10.3%. None of the patients were on a ventilator, were managed without intubation, had an ICU experience, and had no COVID‐19‐related deaths in the entire cohort. A total of 25 cases were accompanied by underlying diseases, of which 5 were in the positive group and 20 were in the negative group. The most common underlying disease in the entire cohort was hypertension, which totaled 8 cases. All patients used PD‐1/PD‐L1 inhibitors, mainly including tirilizumab (44.6%), carilizumab (29.5%), etc. The results of Fisher's exact test and chi‐square test showed that the baseline characteristics of the patients in both the positive and negative groups were similar in terms of cancer tissue type, metastasis, gender, ECOG, underlying disease, PD‐1/PD‐L1 inhibitor type, etc., were similar (*p* > 0.05).

**TABLE 1:** Basic characteristics of patients for the entire study cohort.

Characteristics	Overall	COVID‐19 positive	COVID‐19 negative	*p‐*value
Number of patients, *n* (%)	224	32 (14.3)	192 (85.7)	–
Gender, *n* (%)	224	32	192	0.813
Male	179 (79.9)	25 (78.1)	154 (80.2)	–
Female	45 (20.1)	7 (21.9)	38 (19.8)	–
Stage, *n* (%)	224	32	192	0.503
Metastasis	145 (64.7)	18 (56.3)	127 (66.1)	–
No metastasis	68 (30.4)	12 (37.5)	56 (29.2)	–
Metastases unknown	11 (4.9)	2 (6.2)	9 (4.7)	–
ECOG Score, *n* (%)	224	32	192	1
1	214 (95.5)	31 (96.9)	183 (95.3)	–
2	10 (4.5)	1 (3.1)	9 (4.7)	–
Underlying disease, *n* (%)	25	5	20	0.17
Hypertension	8 (32.0)	1 (20.0)	7 (35.0)	–
Diabetes	1 (4.0)	0	1 (5.0)	–
Hepatitis B	1 (4.0)	1 (20.0)	0	–
Hypertension and cerebral infarction	1 (4.0)	1 (20.0)	0	–
Hypertension and hypothyroidism	1 (4.0)	0	1 (5.0)	–
Hypertension and diabetes	1 (4.0)	0	1 (5.0)	–
Hypertension and hepatitis B	2 (8.0)	1 (20.0)	1 (5.0)	–
Others	10 (40.0)	1 (20.0)	9 (45.0)	–
Cancer types, *n* (%)	224	32	192	0.297
Lung cancer	57 (25.4)	7 (21.9)	50 (26.1)	0.827
Liver cancer	70 (31.3)	8 (25.0)	62 (32.3)	0.537
Cancer of digestive system	62 (27.7)	8 (25.0)	54 (28.1)	0.833
Cancer of urinary system	5 (2.2)	1 (3.1)	4 (2.1)	0.541
Nasopharyngeal carcinoma	7 (3.1)	1 (3.1)	6 (3.1)	1
Others	23 (10.3)	7 (21.9)	16 (8.3)	0.029
Type of PD‐1/PD‐L1 inhibitors, *n* (%)	224	32	192	0.66
Durvalumab	1 (0.4)	0	1 (0.5)	–
Nivolumab	4 (1.8)	1 (3.1)	3 (1.6)	–
Toripalimab	8 (3.6)	0	8 (4.2)	–
Tislelizumab	100 (44.6)	17 (53.1)	83 (43.2)	–
Sintilimab	45 (20.1)	5 (15.6)	40 (20.8)	–
Camrelizumab	66 (29.5)	9 (28.2)	57 (29.7)	–

### 3.2 irAEs in the cohort

We counted the number of irAEs per case in the entire cohort (Table 2), which showed that the number of cases with irAE = 0 was 119 (53.1%), the number of cases with irAE = 1 or 2 was 90 (40.2%), and the number of cases with irAEs ≥3 was 15 (6.7%). In the positive group, the number of cases with irAE = 0 was 18 (56.3%), the number of cases with irAEs = 1 or 2 was 13 (40.6%), and the number of cases with irAEs ≥3 was 1 (3.1%). Fisher's exact test did not reveal any statistically significant disparity in the incidence of irAEs between the positive and negative groups (*p* > 0.05).

**TABLE 2:** irAEs in the queue.

Variables	Overall	irAE in COVID‐19 positive	irAE in COVID‐19 negative	*p*‐value
Number of irAEs, *n* (%)	224	32	192	0.866
0	119 (53.1)	18 (56.3)	101 (52.6)	0.849
1–2	90 (40.2)	13 (40.6)	77 (40.1)	1
≥3	15 (6.7)	1 (3.1)	14 (7.3)	0.702
Type of irAE, *n* (%)	105	14 (13.3)	91 (86.7)	–
Hypoproteinemia	2 (1.9)	1 (7.1)	1 (1.1)	0.25
Nausea and vomiting	15 (14.3)	2 (14.3)	13 (14.3)	1
Fatigue	1 (1.0)	0	1 (1.1)	1
Abdominal discomfort/diarrhea	30 (28.6)	4 (28.7)	26 (28.6)	1
Abnormal liver function	2 (1.9)	0	2 (2.2)	1
Drug‐induced liver injury	2 (1.9)	0	2 (2.2)	1
Myelosuppression	33 (31.4)	3 (21.4)	30 (33.0)	0.541
Hypothyroidism	2 (1.9)	0	2 (2.2)	1
Poor appetite	8 (7.6)	1 (7.1)	7 (7.7)	1
Anemia	3 (2.9)	0	3 (3.3)	1
Arrhythmology	7 (6.7)	3 (21.4)	4 (4.4)	0.048

In addition, we also counted the types of irAE in the whole cohort. If there were multiple irAEs in a case, the irAEs with the highest grade were counted, and there were no irAE‐related deaths in the cohort. The statistical results showed that there were 105 cases of irAEs in the whole cohort, including myelosuppression in 33 cases (31.4%), abdominal discomfort in 30 cases (28.6%), nausea and vomiting in 15 cases (14.3%), followed by nausea and vomiting in 8 cases (7.6%), cardiac arrhythmia in 7 cases (6.7%), hypoproteinemia, hepatic function abnormality, pharmacological hepatic injury, hypothyroidism in 2 cases (1.9%), anemia in 3 cases (2.9%), and 1 case (0.9%) developed malaise. In the positive group, there were 14 cases of irAEs, of which the most common was abdominal discomfort in 4 cases (28.6%), followed by myelosuppression and cardiac arrhythmia, both in 3 cases (21.4%), nausea and vomiting in 2 cases (14.3%), and the presence of hypoproteinemia and nausea in 1 case (7.1%). The results of Fisher's exact test showed that the irAE classes were similar between the two groups except for arrhythmia, which had a statistical difference between the positive and negative groups (*p* = 0.048). In addition, there were 91 cases (47.4%) of irAEs in the negative group, and 14 cases (43.8%) of irAEs in the positive group, and the incidence of adverse reactions was slightly higher in the negative group than in the positive group, but the difference was not statistically significant.

### 3.3 Subgroup analysis after matching

After 1:2 PSM for age, gender, cancer stage/metastasis status, underlying disease, cancer type, PD‐1/PD‐L1 inhibitor type, and ECOG in this cohort, we chose 31 pairs (93 cases) of successfully matched cases for further analysis (Table 3). The difference between the matched groups was only whether they were infected with COVID‐19 or not, and the two subgroups in terms of cancer type, stage/metastasis, ECOG, underlying disease, type of PD‐1/PD‐L1 inhibitor or whether to combine treatments were not statistically different (Fisher's exact test, *p* > 0.05). Comparing the two subgroups of positive and negative (Table 4), we observed that the number and type of irAEs were not significantly associated with whether or not they were infected with COVID‐19 (Fisher's exact test, *p* > 0.05). The incidence of irAEs in the negative group (58.1%) was slightly higher than in the positive group (45.1%) but not statistically significant.

**TABLE 3:** Baseline data of successfully matched medical records.

Characteristics	Overall	COVID‐19 positive	COVID‐19 negative	*p‐*value
Number of patients, *n* (%)	93	31 (33.3)	62 (66.7)	–
Gender, *n* (%)	93	31	62	0.029
Male	73 (78.5)	24 (77.4)	49 (79.0)	–
Female	20 (21.5)	7 (22.6)	13 (21.0)	–
Stage, *n* (%)	93	31	62	0.393
Metastasis	57 (61.3)	17 (54.8)	40 (64.5)	–
No metastasis	33 (35.5)	12 (38.7)	21 (33.9)	–
Metastases unknown	3 (3.2)	2 (6.5)	1 (1.6)	–
ECOG Score, *n* (%)	93	31	62	0.333
1	92 (98.9)	30 (96.8)	62 (100)	–
2	1 (1.1)	1 (3.2)	0	–
Underlying disease, *n* (%)	11	4 (36.4)	7 (63.6)	0.288
Hypertension	3 (27.3)	1 (25.0)	2 (28.6)	–
Diabetes	0	0	0	–
Hepatitis B	1 (9.1)	1 (25.0)	0	–
Hypertension and cerebral infarction	1 (9.1)	1 (25.0)	0	–
Hypertension and hypothyroidism	0	0	0	–
Hypertension and diabetes	0	0	0	–
Hypertension and hepatitis B	0	0	0	–
Others	6 (54.5)	1 (25.0)	5 (71.4)	–
Cancer types, *n* (%)	93	31	62	0.688
Lung cancer	15 (16.1)	7 (22.6)	8 (12.9)	0.246
Liver cancer	28 (30.1)	8 (25.8)	20 (32.2)	0.634
Cancer of digestive system	27 (29.0)	8 (25.8)	19 (30.6)	0.809
Cancer of urinary system	5 (5.4)	1 (3.2)	4 (6.5)	0.662
Nasopharyngeal carcinoma	5 (5.4)	1 (3.2)	4 (6.5)	0.662
Others	13 (14.0)	6 (19.4)	7 (11.3)	0.347
Type of PD‐1/PD‐L1 inhibitors, *n* (%)	93	31	62	0.201
Durvalumab	0	0	0	–
Nivolumab	2 (2.2)	1 (3.2)	1 (1.6)	–
Toripalimab	6 (6.5)	0	6 (9.7)	–
Tislelizumab	42 (45.2)	17 (54.8)	25 (40.3)	–
Sintilimab	22 (23.6)	5 (16.2)	17 (27.4)	–
Camrelizumab	21 (22.5)	8 (25.8)	13 (21.0)	–
Combination therapy, *n* (%)	93	31	62	0.783
Yes	76 (81.7)	26 (83.9)	50 (80.6)	–
No	17 (18.3)	5 (16.1)	12 (19.4)	–

**TABLE 4:** irAEs in the medical record queue were successfully matched.

Variables	Overall	irAE in COVID‐19 positive	irAE in COVID‐19 negative	*p*‐value
Number of irAEs, *n* (%)	93	31 (33.3)	62 (66.7)	0.344
0	43 (46.2)	17 (54.9)	26 (41.9)	0.275
1–2	42 (45.2)	13 (41.9)	29 (46.8)	0.825
≥3	8 (8.6)	1 (3.2)	7 (11.3)	0.261
Type of irAE, *n* (%)	50	14 (28.0)	36 (72.0)	–
Hypoproteinemia	2 (4.0)	1 (7.1)	1 (2.8)	0.486
Nausea and vomiting	7 (14.0)	2 (14.3)	5 (13.9)	1
Fatigue	1 (2.0)	0	1 (2.8)	1
Abdominal discomfort/diarrhea	15 (3.0)	4 (28.6)	11 (30.6)	1
Abnormal liver function	2 (4.0)	0	2 (5.6)	1
Drug‐induced liver injury	2 (4.0)	0	2 (5.6)	1
Myelosuppression	12 (24.0)	3 (21.4)	9 (25.0)	1
Hypothyroidism	0	0	0	–
Poor appetite	3 (6.0)	1 (7.1)	2 (5.6)	1
Anemia	2 (4.0)	0	2 (5.6)	1
Arrhythmology	4 (8.0)	3 (21.4)	1 (2.8)	0.061

**FIGURE 2:**
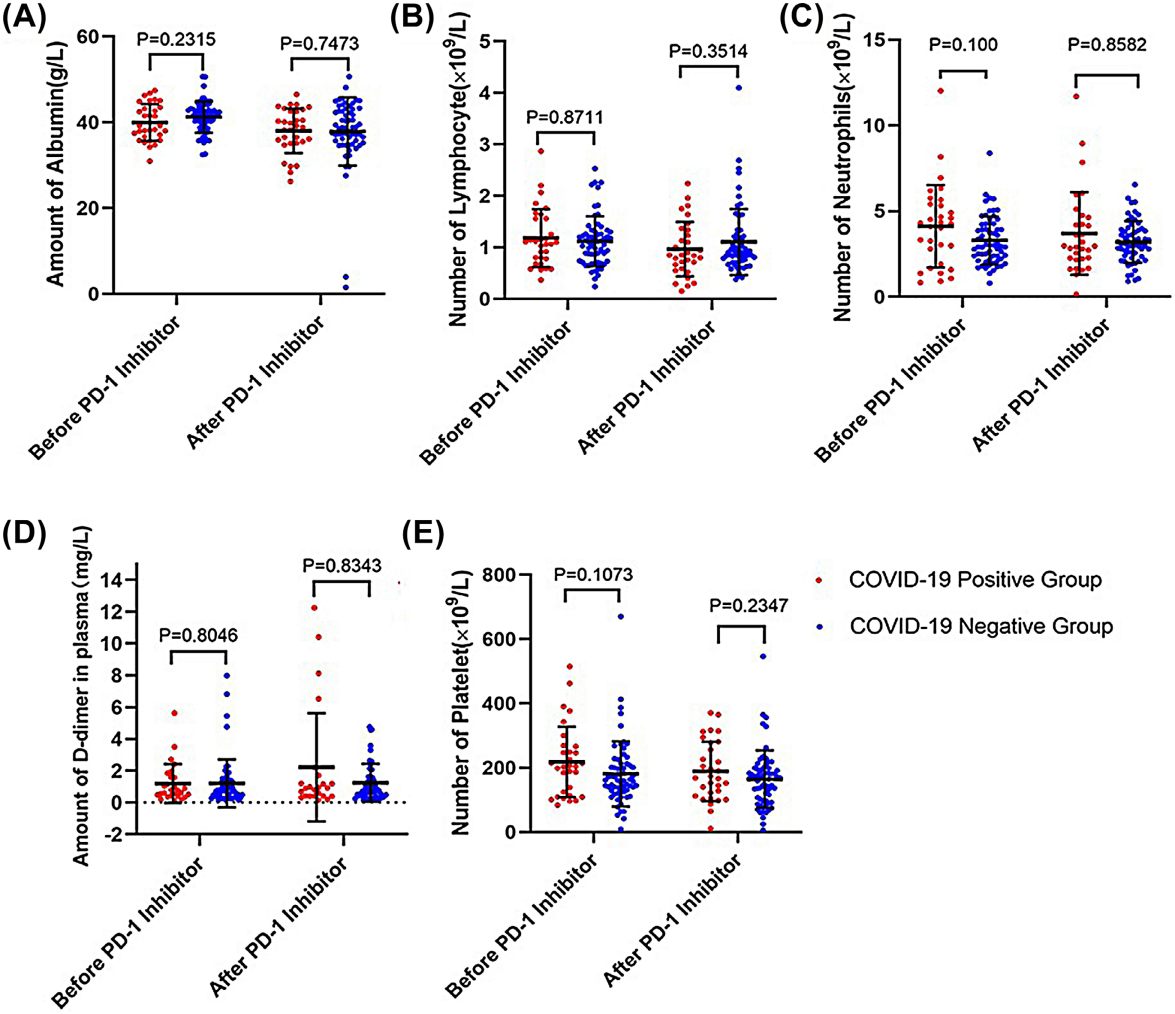
Hematology data after cohort matching (A) albumin count (B) lymphocyte count (C) neutrophil count (D) D‐dimer count (E) platelet count.

**FIGURE 3:**
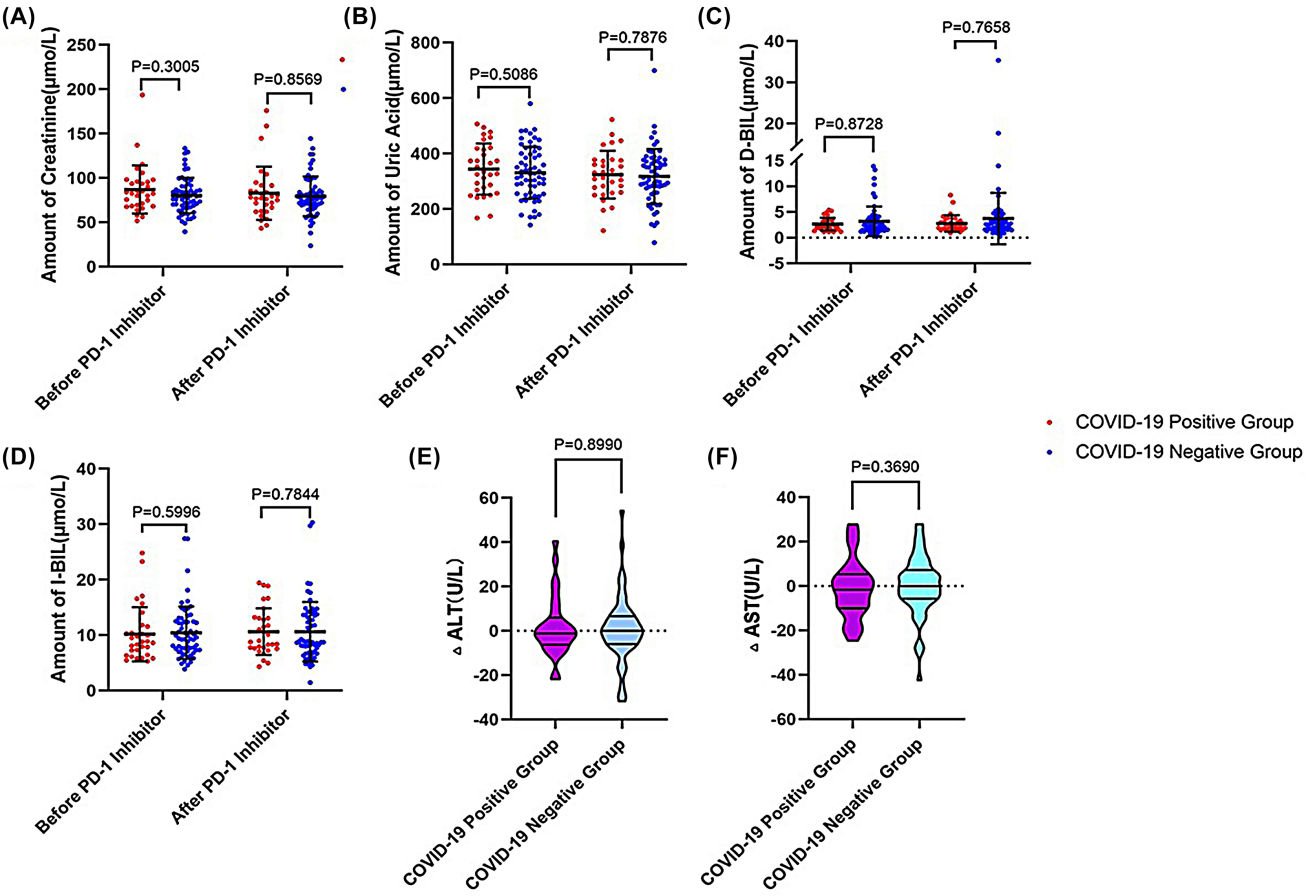
Liver and kidney function data after cohort matching (A) creatinine count (B) uric acid count (C) D‐BIL count (D) D‐dimer count (E) difference between pre‐ and post‐observation of ALT (F) difference between pre‐ and post‐observation of AST.

In addition, to further assess the impact of COVID‐19 infection on the safety of PD‐1/PD‐L1 inhibitor use in our cohort, we analyzed the blood, liver, and kidney function data of patients before and after the use of PD‐1/PD‐L1 inhibitors to assess whether the toxic side effects caused by drug to the cancer patients after infection with COVID‐19 occurred a significant effect. We assessed the immune function differences and liver function changes in the positive and negative subgroups before and after the use of the drug between the two matched groups. The results of the analysis showed (Figure 2) that before and after the use of PD‐1 inhibitors, the number of albumin, lymphocytes, platelets, neutrophils, as well as creatinine, indirect bilirubin, direct bilirubin, and uric acid (Figure 3) in these subgroups were not significant difference (*p* > 0.05), and ALT difference and AST difference were also not significant (*p* > 0.05).

All of the above analyses showed that in the cohort of this study, infection with COVID‐19 had no significant effect on the occurrence of immune‐related adverse reactions, changes in immune function, and changes in liver function in cancer patients using PD‐1/PD‐L1 inhibitors.

### 3.4 Subgroup analysis after matching in different cancer types

In order to more comprehensive studies on COVID‐19 infections in patients with different cancer using PD‐1/PD‐security, the influence of L1 inhibitors we respectively in patients with lung cancer, liver cancer, stomach cancer in the queue for sex, cancer stage/transfer status, basic diseases, PD‐1/PD‐L1 inhibitor types, ECOG 1:2 tend to score matching. Finally succeeded in lung cancer patients received five positive patients and 10 counterparts of negative patients, got six positive in patients with liver cancer patients and 12 patients with negative counterparts of, in patients with gastric cancer received two patients with positive and four negative counterparts patients. The results of the respective analysis of the above three cancers showed that the samples selected from the three cohorts were not significantly different in terms of matching covariates such as gender and stage (Fisher's exact test, *p* > 0.05). There was no significant difference in the number and type of irAEs between COVID‐19 positive and negative subgroups (Fisher's exact test, *p* > 0.05) (Tables **S1**–S3).

## 4 DISCUSSION

PD‐1/PD‐L1 monoclonal antibodies (mAbs) are ICIs that boost T cells' ability to recognize and kill tumor cells.^13^ Moreover, expressed by various cells, PD‐1/PD‐L1 mediates immune regulation through different mechanisms, thus affecting several tumor types, including gastric, bladder, colorectal, and non‐small cell lung carcinoma.^14^, ^15^, ^16^ However, during the course of action, PD‐1/PD‐L1 mAbs may cause T cells to attack healthy tissues other than cancer cells during their course of action, resulting in toxic side effects and posing a risk to patient health. Zhao et al. found that the overall incidence of fatal adverse events with PD‐1/PD‐L1 inhibitors was 0.43% [95% CI 0.25%–0.66%], with the respiratory system mortality being the highest (46.2%).^17^ As for the irAEs associated with the use of immunosuppressants, the most common adverse effects were anemia, rash, enteritis, and thyroid dysfunction.^18^ This also aligns with the patient‐related adverse reactions data collected in our study.

Meanwhile, COVID‐19 infection itself may affect the function of the immune system,^19^ which in turn affects the efficacy and safety of PD‐1/PD‐L1 inhibitors. Moreover, the most typical site of a COVID‐19 attack is the lungs, and combined with the fact that the respiratory system has the highest mortality rate among the lethal adverse events observed in the above studies, the patients using PD‐1/PD‐L1 mAbs during COVID‐19 infection may pose a greater health risk. Theoretically, infection with COVID‐19 in cancer patients undergoing treatment with the drug may simultaneously promote over‐activation of the immune response. This could potentially lead to an increase in irAEs and exacerbate the toxic side effects of immunosuppressants, compromising the outcome of the patient's treatment and life and damaging overall health. However, there is limited and inconclusive data regarding the use of drug in COVID‐19‐infected cancer patients and insufficient evidence to suggest that COVID‐19 infection directly impacts the safety of the PD‐1/PD‐L1 inhibitor therapy. The results of our study showed that there were no statistical differences (*p* > 0.05) in the type of cancer tissue, metastasis, gender, ECOG, underlying disease, type of PD‐1/PD‐L1 inhibitor, and number and type of irAEs between the two conditions of both positive and negative COVID‐19 infection in the entire cohort of patients with the drug. Still, there was a statistical difference in arrhythmia (*p* = 0.048). The results of negative and positive patient tests in the matched cohort were *p* = 0.061. Arrhythmia, as one of the adverse effects after the use of the drug, is mainly manifested as palpitations, chest discomfort, and the electrocardiogram may show extension of the PR interphase, bundle‐branch block, etc.^20^ This result ties well with the previous study wherein cardiotoxicity^21^ and the results of the matched cohort in this study need to be explored in further detail. After conducting 1:2 PSM for age, gender, cancer staging/metastasis, underlying disease, cancer type, PD‐1/PD‐L1 inhibitor type, and ECOG in the cohort of this study, there were no significant differences in the indices of blood routine examination and liver function between the groups before and after the use of the drug (*p* > 0.05), which was similar to Luo et al. reported.^12^ In order to explore whether COVID‐19 infection has different safety effects on PD‐1/PD‐L1 inhibitors in different cancer patients, we further selected patients with larger sample sizes of lung cancer, liver cancer, and gastric cancer for matching analysis. Our finding that COVID‐19 infection did not significantly affect the incidence of immune‐related adverse effects of PD‐1/PD‐L1 inhibitors in patients with cancer in any of these three cancer types is consistent with our results in the overall cohort and suggests the potential value of our findings for patients with different cancers in the clinic. We reviewed the basic characteristics of the patients in this study cohort. Based on the recorded case, it was observed that the patients in this cohort who tested positive for COVID‐19 did not exhibit any symptoms of respiratory distress syndrome. Additionally, none of the patients required intubation, the use of a ventilator, or admission to the ICU. Furthermore, there were no reported fatalities. This result may be explained by the fact that the majority of patients in this cohort had mild cases of the virus. For the clinical typology of the patients, we referred to the Novel Coronavirus Infection Diagnosis and Treatment Protocol (Trial 10th Edition) issued by the National Health Commission. Therefore, our results may be more applicable to patients with mild COVID‐19 infection and can provide greater reassurance regarding the safety of the drug for these individuals. However, further studies are needed to discuss the findings in patients with moderate or severe disease. In addition, with the change of COVID‐19 strain throughout the epidemic, it appears to be in the direction of less virulence but increased in epidemicity. Therefore, taking into account the current virulence of COVID‐19 and its future trends, our findings may be particularly relevant to the current clinical situation of most of these patients with mild COVID‐19 infection.

Some limitations of the current studies are as follows. Firstly, this is a single‐center trial with limited cases and a single data source, resulting in selection bias and weak representativeness. However, we utilized PSM to enhance comparability among individual cases, improve effectiveness, as well as decrease data bias and confounding due to confounding factors.^22^ Second, the retrospective cohort study we used typically results in less accurate evidence compared to prospective randomized clinical trials. Still, our study utilized clinical test data from patients during their hospital stay, which greatly reduced the presence of recall bias typically found in retrospective studies, making the results more credible. Third, several studies have shown that hematological neoplasms are also a risk factor associated with negative outcomes in patients with SARS‐CoV‐2.^23^, ^24^ The present study did not explore rarer cancers such as hematological neoplasms, Merkel cell carcinoma, and all patients with microsatellite instability‐high solid tumors. Hence, additional research is necessary to ascertain the functional importance of COVID‐19 infection and the impact of anti‐PD‐1/PD‐L1 therapy in these individuals.

## 5 CONCLUSION

Our findings suggest that whether COVID‐19 infection occurs (asymptomatic or mild infection) may have little impact on cancer patients using PD‐1/PD‐L1 inhibitors. However, in the real‐world clinical setting, patient‐specific judgments about whether to discontinue drugs still need to be made, especially in patients with their own heart disease.

## AUTHOR CONTRIBUTIONS

While Jie Liu, Wenyige Zhang, and Jinting Cheng contributed nearly equally to the process of getting our submissions ready, Jie Liu and Wenyige Zhang did not take part in the revision process, whereas Jinting Cheng, Yujie Hu, and Beining Zhang did and contributed more. During the later revision of the manuscript, Beining Zhang and Yujie Hu added more detailed information on the included samples and further refined the type and stage of the tumor. Jinting Cheng and Yujie Hu jointly completed the supplementary data analysis and wrote the corresponding results. Beining Zhang prepared the supplementary materials and completed the analysis of the remaining questions. In addition, Peng Huang, Yujie Hu, and Beining Zhang made great contributions to the manual collection of patient information in the initial stage, and Yujie Hu and Beining Zhang also contributed to the chart materials in the preparation stage of the manuscript. For the deletion of Jiasheng Xu is due to his personal will; he does not need to be signed and he only needs to be thanked in the acknowledgments for his guidance in this study. Hence, to duly recognize Xu's significant input, we intend to incorporate an acknowledgment to express gratitude to Xu for his substantive guidance and contributions pertaining to the conceptualization and orientation of this study. The designated content is as outlined below: “We extend our sincere appreciation to Jiasheng Xu for his invaluable guidance and substantial guidance to the methodology and investigation of this study.”

## FUNDING INFORMATION

This work was supported by the National Natural Science Foundation of China [grant number: 81960571 and 81960468].

## CONFLICT OF INTEREST STATEMENT

There are no conflicts of interest in this study.

## ETHICS STATEMENT

All participants involved in the study signed an informed consent form and the study was approved by the Medical Ethics Committee of the Second Affiliated Hospital of Nanchang University.

## Supporting information


Table S1.

## Data Availability

The data generated in this study are available within the article and its supplementary data files.
