# Supplementary material for: Evaluation of the safety of PD‐1/PD‐L1 inhibitors for immunotherapy in patients with malignant tumors after COVID‐19 infection: A single‐center cohort study
Source: Cancer Med. 2024 Oct 8;13(19):e70202. doi: 10.1002/cam4.70202 (PMC11459677; doi:10.1002/cam4.70202)
Supplement: Supplementary file 1 — Table S1. [file CAM4-13-e70202-s001.docx]

**Supplementary Tables**

**Supplementary Table 1** After successful matching analysis of the patients with lung cancer

| Characteristics | Overall | COVID-19  Positive | COVID-19  Negative | *P* value |
| --- | --- | --- | --- | --- |
| Number of patients,n(%) | 15 | 5(33.3) | 10(66.7) | — |
| Gender,n(%) | 15 | 5 | 10 | 0.571 |
| Male | 13(86.7) | 4(80.0) | 9(90.0) | — |
| Female | 2(13.3) | 1(20.0) | 1(10.0) | — |
| Stage,n(%) | 15 | 5 | 10 | 0.505 |
| Metastasis | 12(80.0) | 5(100.0) | 7(70.0) | — |
| No metastasis | 3(20.0) | 0 | 3(30.0) | — |
| Metastases unknown | 0 | 0 | 0 | — |
| ECOG Score,n(%) | 15 | 5 | 10 | — |
| 1 | 15(100.0) | 5(100.0) | 10(100.0) | — |
| 2 | 0 | 0 | 0 | — |
| Underlying disease,n(%) | 2 | 2(100.0) | 0 | 0.095 |
| Hepatitis B | 1(50.0) | 1(50.0) | 0 | — |
| Hypertension &  Cerebral infarction | 1(50.0) | 1(50.0) | 0 | — |
| Type of PD-1/PD-L1 inhibitors,n(%) | 15 | 5 | 10 | 1 |
| Durvalumab | 0 | 0 | 0 | — |
| Nivolumab | 0 | 0 | 0 | — |
| Toripalimab | 0 | 0 | 0 | — |
| Tislelizumab | 12(80.0) | 4(80.0) | 8(80.0) | — |
| Sintilimab | 0 | 0 | 0 | — |
| Camrelizumab | 3(20.0) | 1(20.0) | 2(20.0) | — |
| Number of irAEs,n(%) | 15 | 5 | 10 | 0.441 |
| 0 | 8(53.3) | 4(80.0) | 4(40.0) | 0.282 |
| 1~2 | 5(33.3) | 1(20.0) | 4(40.0) | 0.600 |
| ≥3 | 2(13.3) | 0 | 2(20.0) | 0.524 |
| Type of irAE,n(%) | 7 | 1 | 6 | — |
| Hypoproteinemia | 1(14.3) | 0 | 1(16.7) | 1 |
| Arrhythmology | 1(14.3) | 1(100.0) | 0 | 0.333 |
| Poor appetite | 2(28.6) | 0 | 2(33.3) | 0.524 |
| Myelosuppression | 1(14.3) | 0 | 1(16.7) | 1 |
| Anaemia | 1(14.3) | 0 | 1(16.7) | 1 |
| Leukopenia | 1(14.3) | 0 | 1(16.7) | 1 |

**Supplementary Table 2** After successful matching analysis of the patients with liver cancer

| Characteristics | Overall | COVID-19  Positive | COVID-19  Negative | *P* value |
| --- | --- | --- | --- | --- |
| Number of patients,n(%) | 18 | 6(33.3) | 12(66.7) | — |
| Gender,n(%) | 18 | 6 | 12 | — |
| Male | 18(100.0) | 6(33.3) | 12(66.7) | — |
| Female | 0 | 0 | 0 | — |
| Stage,n(%) | 18 | 6 | 12 | 1 |
| Metastasis | 9(80.0) | 3(50.0) | 6(50.0) | — |
| No metastasis | 9(20.0) | 3(50.0) | 6(50.0) | — |
| Metastases unknown | 0 | 0 | 0 | — |
| ECOG Score,n(%) | 18 | 6 | 12 | — |
| 1 | 18(100.0) | 6(100.0) | 12(100.0) | — |
| 2 | 0 | 0 | 0 | — |
| Underlying disease,n(%) | 0 | 0 | 0 | — |
| Type of PD-1/PD-L1 inhibitors,n(%) | 18 | 6 | 12 | 1 |
| Durvalumab | 0 | 0 | 0 | — |
| Nivolumab | 0 | 0 | 0 | — |
| Toripalimab | 0 | 0 | 0 | — |
| Tislelizumab | 13(72.2) | 4(66.7) | 9(75.0) | — |
| Sintilimab | 5(22.8) | 2(33.3) | 3(25.0) | — |
| Camrelizumab | 0 | 0 | 0 | — |
| Number of irAEs,n(%) | 18 | 6 | 12 | 0.199 |
| 0 | 7(38.9) | 1(16.7) | 6(50.0) | — |
| 1~2 | 11(61.1) | 5(83.3) | 6(50.0) | — |
| ≥3 | 0 | 0 | 0 | — |
| Type of irAE,n(%) | 11 | 5 | 6 | — |
| Abdominal discomfort/Diarrhea | 8(72.7) | 3(60.0) | 5(83.3) | 1 |
| Arrhythmology | 2(18.2) | 1(20.0) | 1(16.7) | 1 |
| Myelosuppression | 1(9.1) | 1(20.0) | 0 | 0.333 |

**Supplementary Table 3** After successful matching analysis of the patients with gastric cancer

| Characteristics | Overall | COVID-19  Positive | COVID-19  Negative | *P* value |
| --- | --- | --- | --- | --- |
| Number of patients,n(%) | 6 | 2(33.3) | 4(66.7) | — |
| Gender,n(%) | 6 | 2 | 4 | — |
| Male | 6(100.0) | 2(33.3) | 4(66.7) | — |
| Female | 0 | 0 | 0 | — |
| Stage,n(%) | 6 | 2 | 4 | — |
| Metastasis | 6(100.0) | 2(100.0) | 4(100.0) | — |
| No metastasis | 0 | 0 | 0 | — |
| Metastases unknown | 0 | 0 | 0 | — |
| ECOG Score,n(%) | 6 | 2 | 4 | — |
| 1 | 6(100.0) | 2(100.0) | 4(100.0) | — |
| 2 | 0 | 0 | 0 | — |
| Underlying disease,n(%) | 0 | 0 | 0 | — |
| Type of PD-1/PD-L1 inhibitors,n(%) | 6 | 2 | 4 | 1 |
| Durvalumab | 0 | 0 | 0 | — |
| Nivolumab | 0 | 0 | 0 | — |
| Toripalimab | 0 | 0 | 0 | — |
| Tislelizumab | 1(16.7) | 0 | 1(25.0) | — |
| Sintilimab | 4(66.7) | 2(100.0) | 2(50.0) | — |
| Camrelizumab | 1(16.7) | 0 | 1(25.0) | — |
| Number of irAEs,n(%) | 6 | 2 | 4 | 0.199 |
| 0 | 0 | 0 | 0 | — |
| 1~2 | 6(100.0) | 2(100.0) | 4(100.0) | — |
| ≥3 | 0 | 0 | 0 | — |
| Type of irAE,n(%) | 6 | 2 | 4 | — |
| Abdominal discomfort/Diarrhea | 2(33.3) | 0 | 2(50.0) | 0.467 |
| Nausea and vomiting | 1(16.7) | 1(50.0) | 0 | 0.333 |
| Poor appetite | 1(16.7) | 1(50.0) | 0 | 0.333 |
| Myelosuppression | 2(33.3) | 0 | 2(50.0) | 0.467 |
